# The clinical impact of supraclavicular lymph node metastasis in patients with locally advanced esophageal squamous cell carcinoma receiving curative concurrent chemoradiotherapy

**DOI:** 10.1371/journal.pone.0198800

**Published:** 2018-06-11

**Authors:** Yen-Hao Chen, Hung-I Lu, Chien-Ming Lo, Yu-Ming Wang, Shang-Yu Chou, Cheng-Hua Huang, Li-Hsueh Shih, Su-Wei Chen, Shau-Hsuan Li

**Affiliations:** 1 Department of Hematology-Oncology, Kaohsiung Chang Gung Memorial Hospital and Chang Gung University College of Medicine, Kaohsiung, Taiwan; 2 Graduate Institute of Clinical Medical Sciences, College of Medicine, Chang Gung University, Taoyuan, Taiwan; 3 School of Medicine, Chung Shan Medical University, Taichung, Taiwan; 4 Department of Thoracic & Cardiovascular Surgery, Kaohsiung Chang Gung Memorial Hospital and Chang Gung University College of Medicine, Kaohsiung, Taiwan; 5 Department of Radiation Oncology, Kaohsiung Chang Gung Memorial Hospital and Chang Gung University College of Medicine, Kaohsiung, Taiwan; 6 Department of Nursing, Kaohsiung Chang Gung Memorial Hospital, Kaohsiung, Taiwan; 7 Department of Anesthesia, Kaohsiung Medical University Hospital, Kaohsiung, Taiwan; H Lee Moffitt Cancer Center and Research Institute, UNITED STATES

## Abstract

**Background:**

To evaluate the clinical significance of supraclavicular lymph node (SCLN) in patients with locally advanced esophageal squamous cell carcinoma (ESCC) receiving curative concurrent chemoradiotherapy (CCRT).

**Materials and methods:**

We retrospectively analyzed all 369 locally advanced ESCC patients treated with CCRT between 2000 and 2015, including 70 patients with SCLN metastasis and 299 patients without SCLN metastasis.

**Results:**

For these locally advanced ESCC patients treated with curative CCRT, N0-2 were significantly associated with superior progression-free survival (PFS) and overall survival (OS) in univariate and multivariable analyses. However, there were no significant differences in PFS and OS between the SCLN metastasis and non-SCLN metastasis groups; a subgroup analysis also revealed there was no significant differences in PFS and OS between patients with and without SCLN metastasis either in the N0-2 or in the N3 subgroup analysis.

**Conclusions:**

Our study suggests that SCLN metastasis is not a prognostic factor in locally advanced ESCC patients receiving curative CCRT, and that SCLNs should be considered to be regional LNs and treated with curative intent.

## Introduction

Esophageal cancer is the eighth most common cancer worldwide and is characterized by extreme aggression and poor prognosis.[1] Esophageal squamous cell carcinoma (ESCC) accounts for more than 90% of esophageal cancer cases, and is the ninth leading cause of cancer deaths in Taiwan.[2] The majority of ESCC patients have locally advanced disease when they are diagnosed. Patients with a resectable disease who are treated with surgical resection generally have better outcomes; however, more than half of patients with locally advanced disease are clinically unresectable, and concurrent chemoradiotherapy (CCRT) remains the standard of care for inoperable or unresectable non-metastasized patients. Nonetheless, in spite of significant improvements having been made in chemotherapy and radiotherapy, the outcomes of such ESCC patients remain poor.[3–7]

Lymph node (LN) metastasis is one of the most important prognostic factors in cancers in general.[8, 9] In the 7^th^ edition of American Joint Committee on Cancer (AJCC) staging system, LNs located in the esophageal drainage area, such as celiac LNs, paraesophageal LNs, and supraclavicular lymph nodes (SCLNs), are defined as regional LNs.[10] Furthermore, N stages are subclassified based on the absolute number of positive LNs instead of the location of regional LN involvement, and a higher N stage is considered a poor prognostic factor.[11–14] However, some studies suggested that SCLN metastases might be considered as distant metastases rather than regional LN metastases.[15–17] Thus, the presence of SCLN metastasis may be considered to indicate stage IV disease, similar to the presence of visceral organ metastasis, such that patients with such metastasis will consequently be excluded from curative surgery and SCLN dissection may be regarded as unrelated to any survival benefit. However, several studies have shown that patients with SCLN metastasis appear to have a better survival rate than those with visceral organ metastasis.[18–21] Furthermore, the significance of SCLN in most series was evaluated in patients receiving esophagectomy. Meanwhile, the significance of SCLN metastasis in locally advanced ESCC patients receiving curative CCRT remains largely undefined. The aim of the present study, therefore, was to elucidate the role of SCLN metastasis in locally advanced ESCC patients receiving curative CCRT.

## Materials and methods

### Patient selection

The records of a total of 1,045 patients with ESCC who were treated at Kaohsiung Chang Gung Memorial Hospital between January 2000 and December 2015 were retrospectively reviewed. Of these 1,045 ESCC patients, we excluded those patients with a history of second primary malignancy, celiac LN metastasis, and distant metastasis other than SCLN metastasis. After that, only those ESCC patients who received CCRT as a curative treatment were included, and a total of 369 ESCC patients were finally selected. These 369 ESCC patients all had locally advanced status and received CCRT as a curative treatment. Any patients who underwent other therapeutic protocols, such as surgical resection followed by chemotherapy/radiotherapy, palliative chemotherapy or radiotherapy alone, or supportive care, were excluded.

The clinical tumor stage of each case of ESCC was determined by chest computed tomography (CT), endoscopic ultrasonography (EUS), or positron emission tomography (PET) scans. The tumor stages were determined according to the 7^th^ AJCC staging system. Data on the treatments and outcomes of the patients were retrospectively retrieved from clinical medical charts and recorded in an electronic database.

Salvage operation was indicated for patients with resectable persistent or recurrent disease after completing CCRT. Patients underwent a radical esophagectomy with cervical esophagogastric anastomosis (McKeown procedure) or an Ivor Lewis esophagectomy with intrathoracic anastomosis, reconstruction of the digestive tract with gastric tube, and pylorus drainage procedures. Two-field lymph node dissection was performed at the same time.

### Identification and definition of supraclavicular lymph nodes and definition of positive lymph nodes

SCLNs were defined as LNs situated between the inferior belly of the omohyoid muscle posteriorly, the clavicle/upper border of the manubrium anteriorly, and inferiorly and cranially inferior to the lower margin of the cricoid.[22] LNs were considered to be metastasis-positive if, first, they were spherical and larger than 10mm in maximum transverse diameter on CT scan or, second, if they were detected to exhibit focal major 18-fluorodeoxy glucose (^18^F-FDG) uptake compared to normal mediastinal activity according to a PET scan.

### Concurrent chemoradiotherapy planning

For local radiotherapy (RT), a customized thermoplastic immobilization device was used for each patient. Then, all patients underwent CT-based simulation and were treated using the three-dimensional conformal radiotherapy (3D-CRT) technique or intensity-modulated radiotherapy (IMRT) technique using 6- or 10-MV photons. For target delineation, the gross target volume (GTV) was defined as the gross tumor and gross lymph nodes on CT scan and/or PET-CT images. The clinical target volume (CTV) comprehensively covered the whole esophagus, the mediastinal LNs, and the bilateral SCLNs. The planning target volume (PTV) was expanded from the CTV by 1.0–2.0 cm margins in all directions. The total dose to the PTV was 50–50.4 Gy in 25–28 daily fractions. For patients with gross LNs in the supraclavicular area, a boosted dose to the LNs would be added for 10–16 Gy in 5–8 daily fractions.

Chemotherapy was performed concurrently with radiotherapy, and consisted of cisplatin (75mg/m^2^; 4-hour drip) on day 1 and 5-fluorouracil (1000mg/m^2^; continuous infusion) on days 1–4 every 4 weeks. Carboplatin was prescribed instead of cisplatin for patients with creatinine clearance < 60 mL/min.

### Statistical analysis

Statistical analyses were performed using the SPSS 19 software package (IBM, Armonk, NY). The chi-square test, Fisher’s exact test, and *t*-test were used to compare data between the two groups. Progression-free survival (PFS) was calculated from the date of starting treatment of the esophageal cancer to the date of disease progression or death from any cause, and overall survival (OS) was calculated from the date of diagnosis of the esophageal cancer to the date of death as a result of all causes or to the date of the last follow-up.

The estimated PFS and OS were calculated using the Kaplan–Meier method, and the differences between groups were assessed using the log rank test for univariate analysis. Multivariable analyses of the prognostic factors for survival were performed using the Cox proportional hazards model. Variables with P ≤ 0.1 in the univariate analysis were selected for multivariable analysis using the enter method. All tests were two-sided and a P less than 0.05 was considered statistically significant.

### Ethics statement

The retrospective analysis was approved by the Chang Gung Medical Foundation Institutional Review Board (201700721B0). All the methods were carried out in accordance with the approved guidelines, and written informed consent of the patients or their families was not judged necessary for this kind of retrospective study by the Chang Gung Medical Foundation Institutional Review Board.

## Results

### Patient characteristics

Upon retrospective review of our ESCC database, a total of 369 locally advanced ESCC patients who received curative CCRT were identified, including 70 ESCC patients with SCLN metastasis and the other 299 patients without SCLN metastasis. The baseline characteristics did not differ significantly among these two groups, apart from N status (P<0.001) and tumor location (P = 0.001). The SCLN group had a higher rate of N3 status and upper third location compared to the non-SCLN metastasis group. At the time of analysis, the median period of follow-up was for 61.9 months (range: 10.4–206 months) for the 65 survivors and 17.3 months (range: 2.3–206 months) for all 369 patients. The 5-year PFS and OS rates were 2.3% and 13%, respectively; a total of 60 patients (20%) received salvage operation due to resectable persistent or recurrent disease after completing CCRT. The clinicopathological parameters of these patients are shown in Table 1.

**Table 1:** Clinicopathological parameters in 369 locally advanced esophageal squamous cell carcinoma patients with/without supraclavicular lymph node (SCLN) metastasis receiving curative CCRT.

Characteristics	SCLN metastasis group (N = 70)	Non-SCLN metastasis group (N = 299)	P value
Age			
< 60 years	54 (77%)	215 (72%)	0.38
≥ 60 years	16 (23%)	84 (28%)	
Gender			
Male	67 (96%)	291 (97%)	0.48
Female	3 (4%)	8 (3%)	
Performance status			
0–1	59 (84%)	262 (88%)	0.46
2	11 (16%)	37 (12%)	
T status			
1 + 2 + 3	33 (47%)	132 (44%)	0.65
4	37 (53%)	167 (56%)	
N status			
0 + 1 + 2	31 (44%)	262 (88%)	<0.001*
3	39 (46%)	37 (12%)	
Grade			
1	10 (14%)	32 (11%)	0.40
2 + 3	60 (86%)	267 (89%)	
Location			
Upper	35 (50%)	87 (29%)	0.001*
Middle + Lower	35 (50%)	212(71%)	
Salvage operation			
Yes	11 (16%)	49 (16%)	0.89
No	59 (84%)	250 (84%)	
Radiotherapy dose			
50–50.4 Gy	67 (96%)	276 (92%)	0.32
< 50Gy	3 (4%)	23 (8%)	
Cycles of chemotherapy			
1	11 (16%)	31 (10%)	0.21
2	59 (84%)	268 (90%)	
Lower	3 (9%)	95 (26%)	

SCLN: Supraclavicular lymph node; CCRT: concurrent chemoradiotherapy

*Statistically significant.

### Clinical impact of SCLN metastasis in the different N statuses

**Fig 1:**
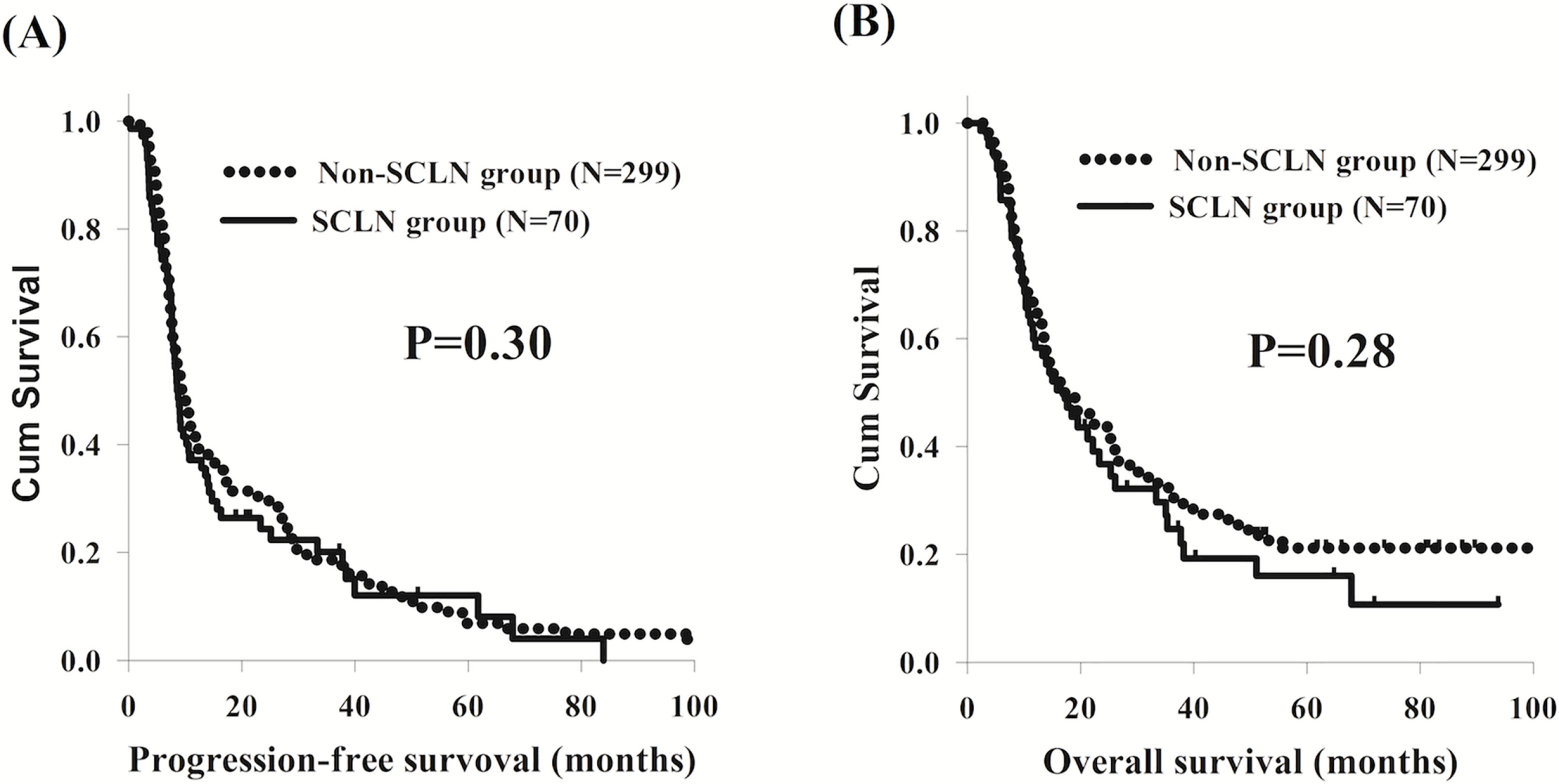
The survival curves of 70 esophageal squamous cell carcinoma patients with supraclavicular lymph node (SCLN) metastasis compared to the 299 patients without SCLN metastasis. (A) Progression-free survival. (B) Overall survival.

**Fig 2:**
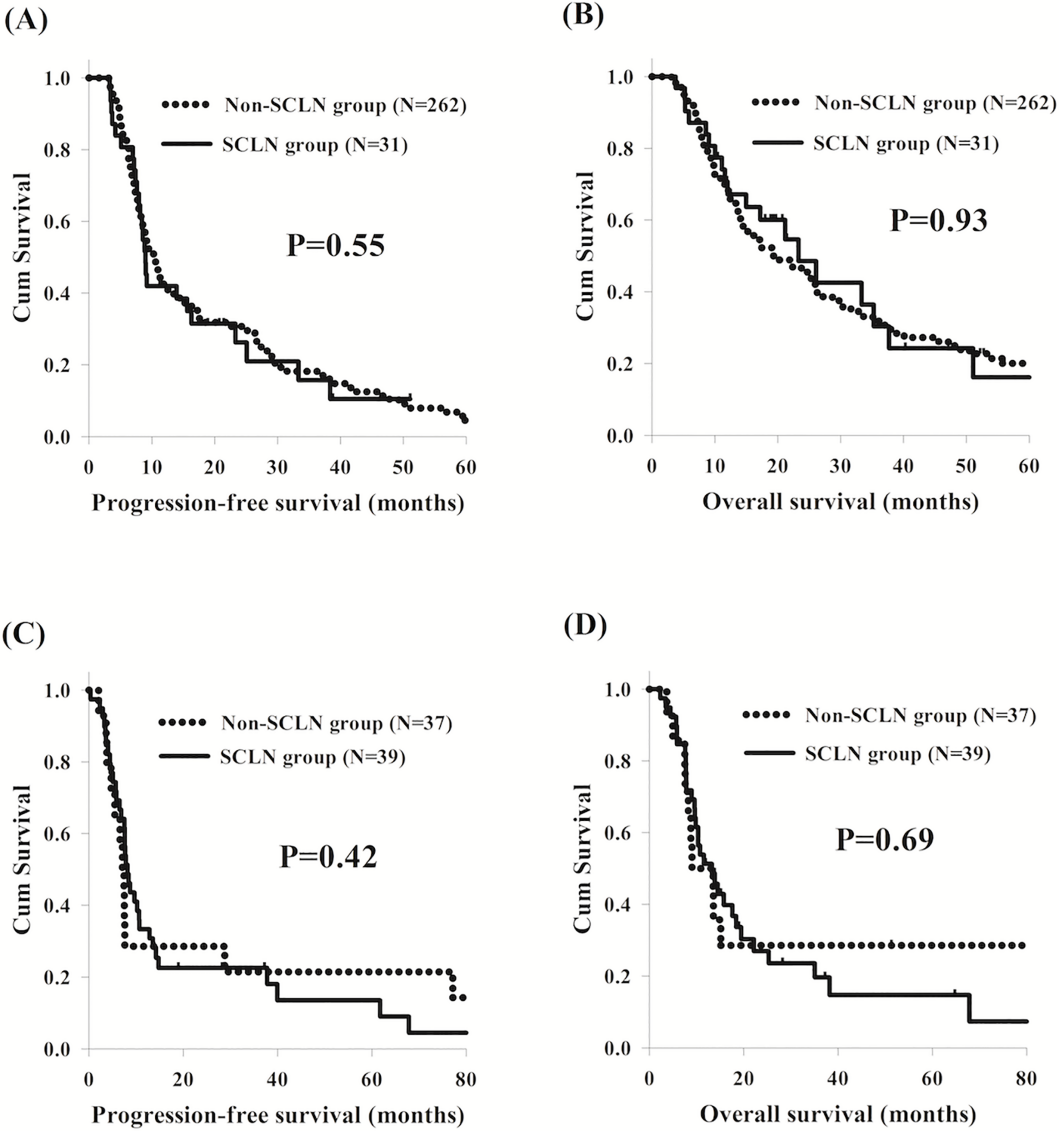
Comparison of survival curves of esophageal squamous cell carcinoma patients with or without SCLN metastasis in different N statuses. (A) N0-2 status, progression-free survival (B) N0-2 status, overall survival. (C) N3 status, progression-free survival (D) N3 status, overall survival. SCLN: supraclavicular lymph node.

In the present study, there were no significant differences in PFS and OS between ESCC patients with or without SCLN metastasis, although SCLN metastasis group had higher percentage of N3 status (Fig 1). In addition, we also found that patients with N3 status had worse PFS and OS than those with N0-2 statuses. Therefore, in order to determine the role of SCLN metastasis in different N status, the 369 ESCC patients were divided into two groups: N0-2 group and N3 group, and then we compared the PFS and OS between the SCLN metastasis group and non-SCLN metastasis group in these two subgroup analyses. Among the 293 patients with N0-2 statuses, who consisted of 31 patients in the SCLN metastasis group and 262 patients in the non-SCLN metastasis group, the survival outcomes were consistent with those of the comparison between the two groups overall (Fig 2A and 2B). For the remaining 76 patients with N3 status, including 39 patients in the SCLN metastasis group and 37 patients in the non-SCLN metastasis group, there were no significant differences in PFS and OS between the two groups (Fig 2C and 2D).

### Survival analysis

In the analysis of PFS, there were no significant differences in terms of tumor grade, tumor location, performance status, SCLN metastasis, salvage operation, radiotherapy dose and cycles of chemotherapy in a univariate analysis. Meanwhile, patients with T1-3 status (P = 0.048), N0-2 status (P = 0.004), age less than 60 years old (P = 0.039) and female patients (P = 0.019) were mentioned to have significantly superior PFS than others. Multivariable analysis revealed that age less than 60 years old (P = 0.009, HR: 0.73, 95% CI: 0.57–0.93), female sex (P = 0.024, HR: 0.46, 95% CI: 0.24–0.90), T1-3 status (P = 0.044, HR: 0.80, 95% CI: 0.65–0.99) and N0-2 status (P = 0.012, HR: 0.72, 95% CI: 0.55–0.93) represented the independent predictive factors of better PFS.

With respect to OS, there were no significant differences in terms of age, gender, tumor grade, tumor location, performance status, SCLN metastasis, salvage operation, radiotherapy dose and cycles of chemotherapy in a univariate analysis. Patients with T1-3 status (P = 0.049) and N0-2 status (P = 0.002) were reported to have better OS than others. According to a multivariable comparison, N0-2 status (P = 0.007, HR: 0.68, 95% CI: 0.52–0.90) represented the independent predictive factors of superior OS. The univariate and multivariable analyses results of PFS and OS for these patients are shown in Table 2.

**Table 2:** Univariate and multivariate analyses results of PFS and OS in in 369 locally advanced esophageal squamous cell carcinoma patients receiving curative CCRT.

Characteristics	No. of patients	Univariate analysis		Multivariable analysis		Univariate analysis		Multivariable analysis	
		Median PFS (months)	P value	HR (95% CI)	P value	Median OS (months)	P value	HR (95% CI)	P value
Age									
< 60 years	269 (73%)	10.2	0.039*	0.73 (0.57–0.93)	0.009*	17.7	0.75		
≥ 60 years	100 (27%)	8.8				19.2			
Gender									
Male	358 (97%)	9.6	0.019*			18.2	0.06		
Female	11 (3%)	17.3		0.46 (0.24–0.90)	0.024*	40.0		0.69 (0.35–1.34)	0.27
T status									
1 + 2 + 3	165 (45%)	11.8	0.048*	0.80 (0.65–0.99)	0.044*	22.3	0.049*	0.83 (0.66–1.04)	0.11
4	204 (55%)	8.8				15.6			
N status									
0 + 1 + 2	293 (79%)	10.9	0.004*	0.72 (0.55–0.93)	0.012*	21.2	0.002*	0.68 (0.52–0.90)	0.007*
3	76 (21%)	7.6				11.8			
Grade									
1	42 (11%)	9.0	0.69			14.8	0.22		
2 + 3	327 (89%)	10.6				19.2			
Location									
Upper	122 (33%)	8.9	0.99			17.3	0.51		
Middle + Lower	247 (67%)	10.1				19.1			
Performance status									
0–1	321 (87%)	9.8	0.65			18.9	0.65		
2	48 (13%)	9.9				14.5			
SCLN metastasis									
Yes	70 (19%)	8.5	0.30			17.2	0.28		
No	299 (81%)	10.3				18.4			
Salvage operation									
Yes	60 (16%)	12.8	0.44			21.2	0.74		
No	309 (84%)	9.3				17.1			
Radiotherapy dose									
50–50.4 Gy	343 (93%)	9.8	0.60			18.2	0.28		
< 50Gy	26 (7%)	9.0				15.7			
Cycles of chemotherapy									
1	42 (11%)	10.9	0.61			16.2	0.98		
2	327 (89%)	9.6				18.3			

CCRT: concurrent chemoradiotherapy; SCLN: supraclavicular lymph node; PFS: progression-free survival; OS: overall survival; HR: hazard ratio; CI: confidence interval

*Statistically significant.

Whether univariate or multivariable analyses, there were no significant differences in PFS and OS between ESCC patients with or without SCLN metastasis.

## Discussion

Patients with SCLN metastasis constitute a small portion of the overall population of patients diagnosed with ESCC. In the 7^th^ AJCC staging system, SCLNs are defined as regional LNs, and N stages are subclassified based on the number of positive LNs.[10] In our study, patients with SCLN metastasis only account for 13% of those who received CCRT as a curative treatment; we also found there were no significant differences in PFS and OS between ESCC patients with or without SCLN metastasis. Nevertheless, SCLNs have been considered to be distant LNs in several studies; therefore, esophageal cancer with SCLN metastasis is commonly regarded as a systemic disease and generally excluded from the indications for curative treatment.[15–17] However, growing evidence has suggested that SCLN metastasis does not compromise prognosis in comparison with other regional LN metastasis.[16, 19, 23] Therefore, the clinical impact of SCLN metastasis remains controversial.

To the best of our knowledge, several studies have evaluated and discussed the effects of SCLN metastasis in relation to the various treatment options and their outcomes. Recently, three-field lymphadenectomy with cervical LN dissection including SCLNs has been performed aggressively in some Asian countries.[15–17, 24] Cho *et al*. showed that SCLN metastasis did not compromise the clinical outcomes in esophageal cancer patients receiving neoadjuvant chemoradiotherapy following surgery.[24] Another study, reported by Zheng *et al*., also revealed that SCLN metastasis was not a poor prognostic factor and that the number of lymph nodes involved was strongly associated with overcall survival.[17] Furthermore, two Japanese studies revealed that SCLN metastasis was not prognostically unfavorable when SCLNs were considered as regional LNs rather than distant LNs, meaning from M1 status (metastatic disease) to M0 status.[16, 25] In our study, we found that SCLN metastasis is not a prognostic factor in locally advanced ESCC patients receiving curative CCRT. Therefore, we suggest that SCLNs should just be considered another type of regional LNs rather than being viewed as distant LNs.

CCRT has an important role in the treatment of esophageal cancer and remains the standard of care for patients with locally advanced ESCC. Several studies have documented that CCRT has beneficial effects on the primary tumors and involved LNs.[26–28] In the CROSS trial, neoadjuvant chemoradiotherapy was found to be capable of decreasing the rate of nodal involvement and increasing the percentage of pathological complete response after treatment.[28] Schneider *et al*. showed that histomorphologic tumor regression and LN status (ypN) were significant prognostic parameters for patients with complete resections following neoadjuvant radiochemotherapy for esophageal cancer.[27] Another study, reported by Donohoe *et al*., found that neoadjuvant chemoradiotherapy supported the survival of esophageal cancer patients with LN metastasis initially but that pathologic N status was negative after treatment.[26] These findings also suggested that neoadjuvant chemoradiotherapy can be effective not only for the primary tumors but also the involved LNs.

In our study, SCLN metastasis was not found to be a prognostic factor. We suggest that this finding in our study and other previous studies may be related to several factors. First, the location of the SCLNs is generally included in the field of radiotherapy, which can be planned for thoracic esophageal cancer. Thus, although SCLNs are regarded as distant LNs in some studies, they are effectively considered regional LNs for the purposes of treatment. Second, the extent of structural damage to a tissue caused by radiotherapy generally depends on cell radiosensitivity. The relationships between anatomical radiation damage and failures of organ function are thus different for different organs. There are no major organs or hollow organs near the SCLNs, meaning that in order to increase the treatment efficacy, the radiotherapy doses delivered to SCLNs can be relatively high, compared to those delivered to the other regional LNs. Therefore, for locally advanced ESCC patients suitable for CCRT with curative intent, SCLNs should be regarded as regional LNs rather than distant LNs.

Several past studies have investigated the relationship between the number of involved LNs and survival outcomes.[12, 13] In our study, higher N status, meaning a greater number of positive LNs, retained statistical significance as an adverse prognostic factor for PFS and OS in univariate and multivariable analyses. In addition, we also found there was no difference in PFS and OS between patients with or without SCLN metastasis in the subgroup analysis, whether for the N0-2 group or N3 group. Tachimori *et al*. also reported the same finding, revealing that SCLN metastasis did not predict survival outcomes in respectively N1, N2, or N3 subgroups.[16] In general, SCLN metastasis is not a prognostic factor in locally advanced ESCC patients who received CCRT, and SCLNs should be considered as regional LNs and treated with curative intent if the number of involved LNs is limited. In addition, SCLNs could tolerate higher dose of radiotherapy due to no major organs or hollow organs nearby, resulting in better treatment efficacy. In clinical practice, CCRT with more aggressive treatment, such as salvage operation, should be performed for these ESCC patients with SCLN metastasis.

This study had several limitations. First, it was a retrospective study of patients treated at a single institution and almost all patients in our study were locally advanced status, so the sample size was relatively small. Second, there were more patients with N3 status and upper locations of the primary tumor in the SCLN metastasis group, but no significant differences in overall survival were found between these two groups. However, to the best of our knowledge, this study, at present, covers the largest series of ESCC patients with SCLN metastasis who underwent curative CCRT and may thus be useful for understanding this rare disease entity.

In conclusion, the results of our study suggest that SCLN metastasis is not a prognostic factor in locally advanced ESCC patients receiving curative CCRT, and that SCLNs should be viewed as regional LNs and treated with curative intent if the number of involved LNs is limited. Further larger prospective studies are warranted in order to clarify the clinical impact of SCLN metastasis in locally advanced ESCC patients receiving curative CCRT.
